# Force Transduction and Lipid Binding in MscL: A Continuum-Molecular Approach

**DOI:** 10.1371/journal.pone.0113947

**Published:** 2014-12-01

**Authors:** Juan M. Vanegas, Marino Arroyo

**Affiliations:** LaCàN, Universitat Politècnica de Catalunya-BarcelonaTech, Barcelona, Spain; University of Bern, Switzerland

## Abstract

The bacterial mechanosensitive channel MscL, a small protein mainly activated by membrane tension, is a central model system to study the transduction of mechanical stimuli into chemical signals. Mutagenic studies suggest that MscL gating strongly depends on both intra-protein and interfacial lipid-protein interactions. However, there is a gap between this detailed chemical information and current mechanical models of MscL gating. Here, we investigate the MscL bilayer-protein interface through molecular dynamics simulations, and take a combined continuum-molecular approach to connect chemistry and mechanics. We quantify the effect of membrane tension on the forces acting on the surface of the channel, and identify interactions that may be critical in the force transduction between the membrane and MscL. We find that the local stress distribution on the protein surface is largely asymmetric, particularly under tension, with the cytoplasmic side showing significantly larger and more localized forces, which pull the protein radially outward. The molecular interactions that mediate this behavior arise from hydrogen bonds between the electronegative oxygens in the lipid headgroup and a cluster of positively charged lysine residues on the amphipathic S1 domain and the C-terminal end of the second trans-membrane helix. We take advantage of this strong interaction (estimated to be 10–13 kT per lipid) to actuate the channel (by applying forces on protein-bound lipids) and explore its sensitivity to the pulling magnitude and direction. We conclude by highlighting the simple motif that confers MscL with strong anchoring to the bilayer, and its presence in various integral membrane proteins including the human mechanosensitive channel K2P1 and bovine rhodopsin.

## Introduction

Mechanosensitive (MS) proteins are responsible for the conversion between mechanical and chemical signals. Mechanical stimuli imposed by cellular membranes on integral membrane proteins have been shown to play an important, and sometimes critical, role in their function. Such mechanical stimuli can be provided by tension, membrane thickness (through hydrophobic mismatch), spontaneous curvature, and stress distribution given by lipid composition or bilayer asymmetry [1]–[3]. Bacterial MS channels act as emergency release valves to prevent cell lysis under hypo-osmotic shock, and have been extensively studied as model systems for larger or more complex channels [2]. The MS channel of large conductance (MscL) has become a favorite in recent years due to its smaller size (94 kDa), the ease of transfer to in vitro systems, and the availability of a high resolution structure [4], [5].

**Figure 1:**
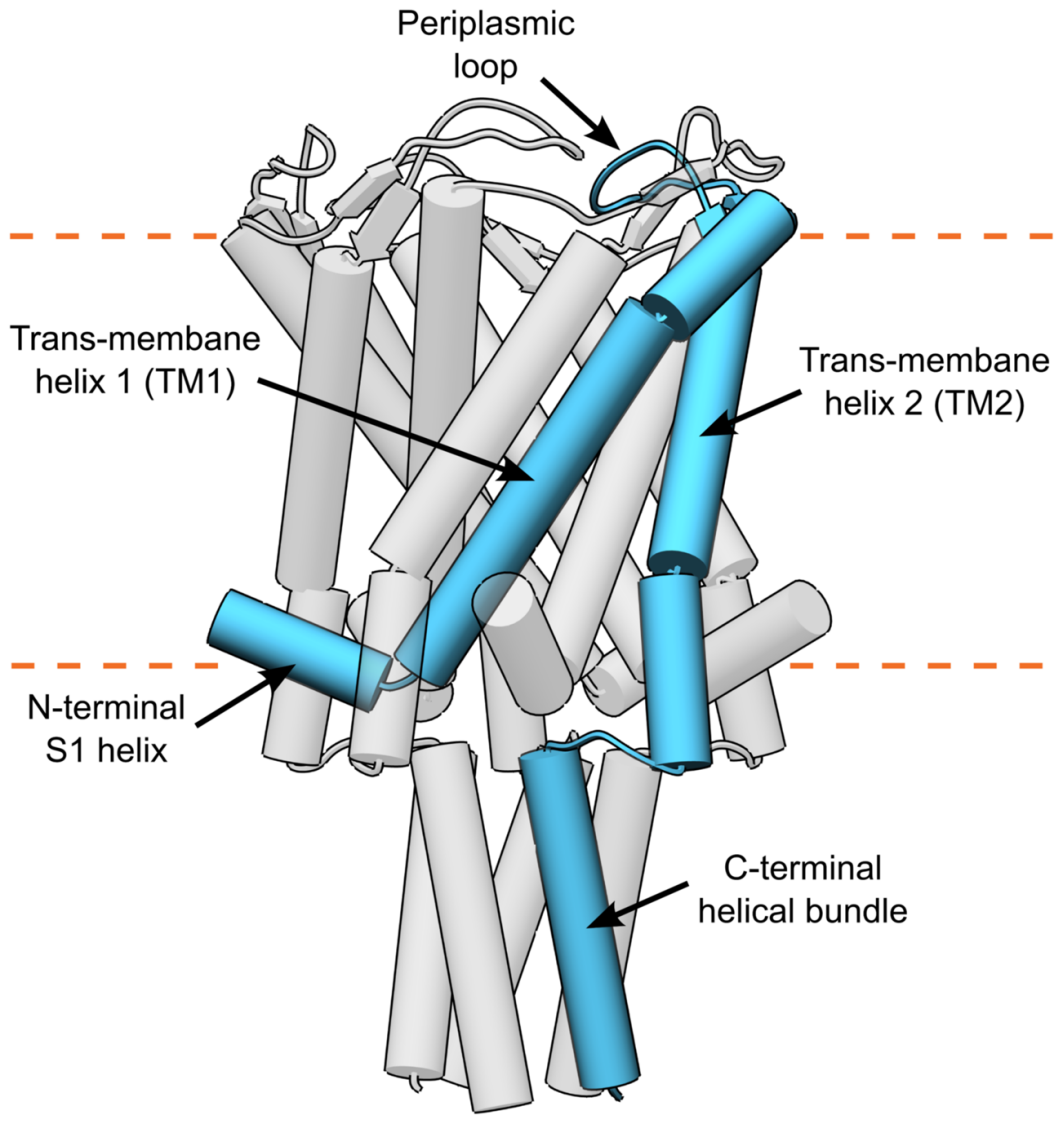
MscL structure. Cartoon representation (alpha-helices shown as cylinders) of the pentameric structure of MscL. A single monomer is highlighted in blue showing the N-terminal S1 helix, the first trans-membrane helix (TM1), the periplasmic loop, the second trans-membrane helix (TM2), and the C-terminal helical bundle.

The primary stimulus for MscL gating is membrane tension [6], although externally added amphipaths, such as lysolipids, can also induce spontaneous opening in the absence of tension [7], [8]. During opening, it is believed that the two trans-membrane (TM) helices in each of the five subunits (see Fig. 1) increase their tilt and move outwardly in the open configuration [1], [2]. However, the physical mechanisms responsible for gating of MscL are not fully understood. The channel opening is most likely the result of many lipid-protein and intra-protein interactions as suggested by numerous mutagenic studies: replacing the small hydrophobic residues that line the pore with hydrophilic amino acids produces channels that spontaneously open in the absence of tension [9], [10], replacement or deletion of amino acids in the N-terminal S1 domain (see Fig. 1) on the cytoplasmic side leads to partial or complete loss of function [11]–[13], and mutations at the rim on the periplasmic side are also poorly tolerated [9], [14]. A large number of these mutations occur at amino acids that are located at the bilayer-protein interface, emphasizing the importance of this region of the protein. Furthermore, observations from a recent molecular dynamics study indicate that the interaction between lipids and hydrophobic amino acids, such as phenyl alanine, play an important role in gating [15]. The molecular surface of proteins is rugged with many features that may serve as binding sites for neighboring lipids [16].

The energetics of the gating process in MscL have been interpreted in terms of a vapor-lock model, where the channel opening is dominated by the hydration of the pore [17] as supported by mutagenesis studies [9], [10], and by models relying on non-specific interactions, which emphasize the mechanical state of the membrane near the bilayer-protein interface [18]–[20]. In the latter model, the transition between open and closed states of MscL is predicted to critically depend on the energetics of the bilayer, which is elastically deformed in the vicinity of the protein due to the hydrophobic mismatch. This conceptual model centered on the membrane is supported by experimental evidence, showing that MscL gating strongly depends on lipid chain length and head group size [7], [8]. Such models, focusing on either the protein or the bilayer, provide insight on MscL gating, but do not account for the full complexity of the process as the bilayer-protein interactions are largely ignored. The specific molecular interactions at this interface have not been sufficiently examined, and may provide clues to integrate the protein and membrane physics involved in MscL gating. In particular, the S1 helical domain, whose amphipathic motif is highly conserved among bacterial species [11], is one such interaction site that needs further investigation. This S1 domain was not resolved in the first released MscL crystal structure [4] (PDBID 1MSL), and was later proposed to form a secondary gate on the aqueous region of the cytoplasmic side of the protein [21], [22]. However, further refinement of the x-ray data resulted in a new structure (PDBID 2OAR) where the S1 domain sits at the lipid water interface with the helix roughly oriented along the membrane plane [5].

Here, we examine the interaction of lipids with the bacterial MscL using atomistic molecular dynamics (MD) simulations. We first focus on the internal stress of the membrane-protein system, since mechanosensing involves transduction of the mechanical state of the membrane into the protein chemistry. Rather than examining the local stress tensor directly, as commonly done [23], [24], we focus on the traction, a more revealing continuum measure of forces derived from the stress tensor. The traction at the protein-lipid interface provides the actual forces per unit area exerted by one sub-system onto the other. Our traction results suggest that membrane tension induces forces that are localized to specific regions of the protein. We find long-lived interactions in these regions of the protein, where positively charged residues provide strong anchoring to the membrane. We characterize and quantify these molecular interactions, and further exploit them to accelerate the channel opening process in steered MD simulations. We present a possible framework to interpret how internal changes in the bilayer mechanics, due to effects such as hydrophobic mismatch or tension, can translate into forces at specific protein sites during gating. Furthermore, our results establish a link between sequence, structure, and force transmission, provide a way to integrate existing gating models that focus on either the protein or the membrane, and incite further studies to ascertain the role of asymmetry and specific lipid-binding motifs in MscL and other integral membrane proteins.

## Results

### Tension and Forces on the Protein Surface

We simulated the bacterial MscL protein from *Mycobacterium tuberculosis* (PDB id 2OAR [5]) embedded in a membrane patch composed of POPE (1-palmitoyl-2-oleoyl-sn-glycero-3-phosphoethanolamine) lipids. The large POPE membrane patch (512 lipids) was first equilibrated for 400 ns, and after embedding of the protein, the entire system was further simulated for 500 ns to reach equilibrium (see Methods). Before diving into the molecular details of the membrane-protein simulation, we first aim to characterize the system from a mechanical perspective.

**Figure 2:**
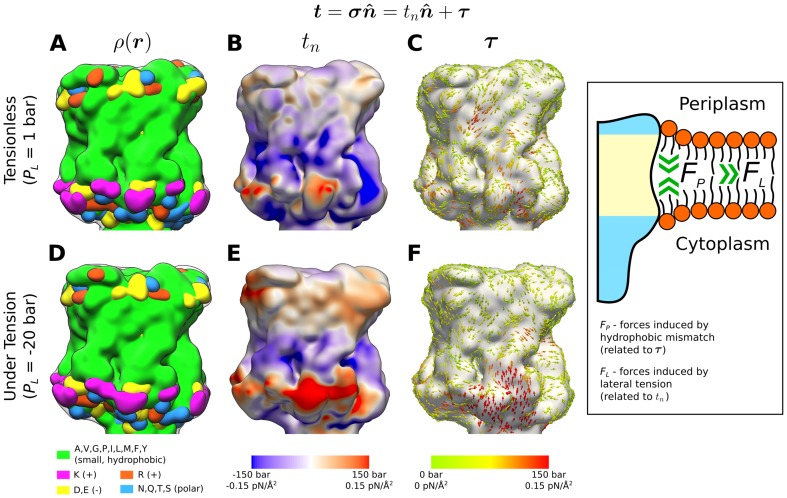
Effects of tension on the surface traction of MscL. The mass-density iso-contours on the left (A and D) show the surface of the protein, which defines the normal unit vector 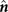. The location of key amino acids at the lipid-water interface for MscL in a tensionless state (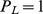 bar) and under tension (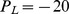 bar) has been highlighted. The traction is calculated from the local stress tensor (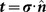, see Methods) and is decomposed into a component along the direction normal to the surface, of magnitude 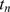, and a tangential component, 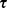, so that 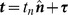. (B) and (C) show 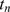 and 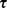 for the tensionless system, and similarly, (E) and (F) show the same quantities for the system under tension. The inset on the right illustrates a conceptual link between 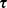 and forces induced by hydrophobic mismatch, 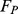, as well as 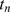 and forces induced by lateral tension, 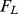.

We begin by analyzing the MD results following a continuum approach that quantifies the internal stress state of the system and how tension affects the interaction between the membrane and the MscL protein. We focus on the traction, a quantity that is best suited to understand the stress at interfaces such as the bilayer-protein interface, and evaluate it on the surface of MscL as shown in Fig. 2. The traction vector (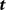, a force per unit area) provides a direct measurement of the forces acting on a given surface within the material, as defined by a unit normal vector 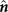. The traction on the protein surface is computed by first calculating the stress tensor (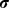, a mathematical device that characterizes the internal forces of a continuous medium) within the simulation volume, and then computing the product of this tensor with 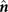 (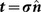) along the surface. The stress tensor was calculated with our recently developed custom version of GROMACS [25] using the central force decomposition methodology [25], [26] over a 100 ns simulation following equilibration (see Methods for details). The traction vector can be decomposed into a normal component, of magnitude 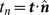, and a shear component, 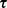, tangential to the surface. The surface of the protein at nominal membrane tension (lateral pressure, 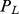, of 1 bar) is shown in Fig. 2A (an iso-contour of the mass-density), which illustrates the location of key amino acids at the lipid-water interface and at the hydrophobic core. The normal and tangential components of the traction for this tensionless simulation are shown in Fig. 2B and C respectively. The normal component of the traction (Fig. 2B) shows a largely asymmetric distribution of forces, with the cytoplasmic region exhibiting localized regions of larger normal traction. Interestingly, there appears to be a correlation between the large positive 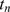 values (pointing away from the surface, i.e., pulling on it) and the location of some of the lysine residues. These large positive normal tractions are balanced by neighboring regions of large negative 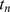 values (pushing on the protein) on the cytoplasmic side, which appear to be unspecific, without a clear correlation to any amino acid. These “pushing” forces may be a result of the strong interactions between the cytoplasmic side of the protein and the lipid tails, as discussed in the next section. The tangential component of the traction (Fig. 2C) does not show an evident localization pattern, although the forces in the hydrophobic core region consistently point towards the bilayer midplane.

While lateral tension induces the opening of the MscL channel, this is a relatively slow process with a timescale in the order of several hundred nanoseconds to microseconds [23], [27]. Therefore, even under large lateral tensions, the membrane-protein system is expected to exist in a quasi-equilibrium state for some time (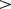
100 ns) before an eventual channel opening event. We take advantage of this, and simulate the system with a large nominal lateral tension (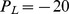 bar, inducing a membrane stretch of 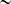
6%) for 140 ns, and characterize the traction on the surface of the protein under these quasi-equilibrium conditions. The starting point for this simulation was the final configuration after 500 ns under normal conditions (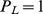 bar), and the system approached a steady state by 40 ns when the projected area in the membrane plane reached a constant value. The protein surface as well as the 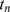 and 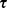 components of the traction for the system under tension are shown in Fig. 2D, E, and F respectively. Although the protein is slightly deformed as the membrane is tensed, it remains in a similar closed conformation. Both components of the traction show a clear asymmetry in this case, with significantly larger forces in regions at the lipid-water interface and particularly at the cytoplasmic side. Similarly to the tensionless case, the regions with the biggest positive 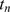 values (Fig. 2E) are largely correlated with the location of lysine residues on the cytoplasmic side. Although the periplasmic side of the protein also shows higher positive 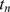 values, these do not appear to be as localized as in the cytoplasmic side.

While the physical interpretation of stress and tractions reaches its limits at the molecular level, our calculations show an orchestrated distribution of forces under tension and at rest, which suggest a correlation between mechanical and chemical features. At a more conceptual level, the traction plots suggest a connection between 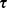 and forces induced by hydrophobic mismatch, 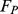, as well as between 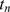 and forces induced by lateral tension, 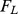, as shown in the inset on the right of Fig. 2. For instance, tension thins down the membrane and therefore increases the hydrophobic mismatch between MscL and the surrounding POPE bilayer, which would in turn increase 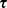. Yet, real systems may depart from this idealized view depending on specific features in each case. For instance, MscL in our simulations appears to be under a small positive hydrophobic mismatch in the POPE membrane, which is partially compensated by local deformation of the bilayer. However, the mismatch also induces a slight tilt of the protein (<10°) with respect to the bilayer normal, as has been observed for helical peptides [28]–[30] and suggested for helical portions of larger channels such as KcsA [31].

### Molecular Interactions at the Bilayer-Protein Interface

**Figure 3:**
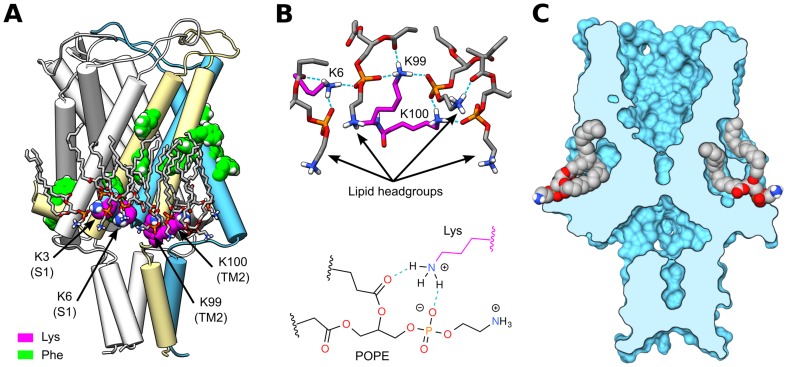
Lipid binding interactions on the cytoplasmic side of MscL. (A) Cartoon representation of MscL highlighting all the Phe (green) and cytoplasmic Lys (magenta) amino acids in two of the protein monomers, highlighted in light yellow and blue. For clarity, one monomer (blue) shows only the lysines on the S1 domain (K3 and K6), and the adjacent monomer (yellow) shows only the lysines on TM2 (K99 and K100). Lipids that hydrogen-bond the selected lysines are shown in gray. (B) Common hydrogen-bonding pattern observed between the oxygens in the negatively-charged portion of the lipid headgroup and the positively-charged Lys. (C) Lipid tails of tightly-bound lipids accommodate in cavities as shown in a vertical cross section of a surface representation of MscL.

The traction results presented in the previous section prompt a close examination of the molecular interactions at the bilayer-protein interface in the headgroup regions, and particularly to the Lys-POPE interactions on the cytoplasmic side. The majority of lysines in MscL are localized at the cytoplasmic side of the protein. Each of the five subunits contains two Lys residues (K3 and K6) on the amphipathic S1 helix at the N-terminus, a Lys (K33) at the first trans-membrane helix (TM1) that lines the channel pore, and two others (K99 and K100) at the C-terminal end of the second TM helix (TM2). The cytoplasmic lysines (K3, K6, K99, and K100) are in close spatial proximity to each other and directly contact the lipid headgroups (see Fig. 3A). Examination of all the lipids that hydrogen-bond the protein reveals a large number on the cytoplasmic side, 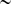
40 at any given time, in contrast to the periplasmic side where this number is reduced by half (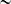
21 at any given time). The majority of lipids (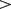
85%) that hydrogen-bond the protein on the cytoplasmic side do so with the four lysines as shown in Fig. 3A, where for clarity, K3 and K6 are shown in one monomer and K99 and K100 in the adjacent monomer. The remaining lipids on this side of the protein hydrogen-bond the backbone atoms or other polar residues such as threonine, asparagine, and glutamic acid. The Lys amine group hydrogen-bonds the oxygens in the phosphate or carbonyl portions of the lipid headgroup, and often interact with more than one oxygen of the same lipid forming a “clamp” pattern as shown in Fig. 3B. We characterize the dynamic nature of these hydrogen-bonding interactions by measuring the distance between the lipid phosphorus to the Lys sidechain nitrogen (Fig. S1), which has an average value of 0.4 nm when the lipid phosphate oxygens hydrogen-bond a lysine. Time traces of the P-N distance for 10 selected “tightly-bound” lipids (Fig. S1) show that many of these Lys-POPE interactions are established within the first few nanoseconds and continue for the duration of the 500 ns period, while others come into place later in the simulation or are only present intermittently (100–300 ns).

In addition to the electrostatic interactions, some of the tightly-bound Lys hydrogen-bonded lipids further increase their interaction with the protein by accommodation of the lipid tails within hydrophobic cavities (Fig. 3C) that are largely surrounded by Phe residues (Fig. 3A). These cavities appear to preferentially select the 16 carbon chain of the lipid versus the longer 18 carbon chain (the shorter tail is inserted in 8 out of 10 lipids), allowing for one lipid chain to be inserted into the protein cavity while the other chain is “free”. This observation is in good agreement with fluorescence quenching experiments that show increased affinity of lipids to the cytoplasmic side of MscL, as well as preference for lipids with 16 carbon chains [32]. On average, 10 lipids (2 per monomer) appear to be tightly-bound to the protein, presenting both Lys-heagroup hydrogen-bonding as well as tail insertion into the protein cavities. Time-based analysis (Fig. S2) shows that the lipid-protein association occurs early on in the simulation (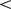
50 ns), and the tightly-bound lipids remain associated to the protein throughout the simulation. Quantification of the individual molecular volume of the lipids (see Methods) displays a rapid decrease due to the reduced mobility of the tails inside the cavities (Fig. S2A). Similarly, the variance in the distance between individual lipids and the center of mass of the protein shows that the tightly-bound lipids stay at a fixed distance from the protein, while the remaining lipids continuously drift away as expected from random lateral diffusion (Fig. S2B). Put together, all these data show a well organized assembly of 10 tightly-bound lipids, which intimately interact with the protein and exert localized forces on it through hydrogen bonds with lysines at the cytoplasmic side.

Examination of the hydrogen-bonding at the periplasmic side of the protein does not reveal a clear pattern comparable to that described in the cytoplasmic side of MscL. However, POPE lipids are often found forming hydrogen bonds with serine, asparagine, arginine, and glutamine protein residues, as well as the backbone atoms of the periplasmic loop. These lipids have long residence times near the protein and tend to diffuse with it as has been previously reported in other membrane channels [33].

### Estimating the Strength of Lipid Binding

Although the interaction between the tightly-bound lipids and the protein is complex, with multiple sites and geometric confinement of the lipid tails, we expect that the energetics of binding are dominated by the hydrogen bonds between the lysines and the lipid oxygens. We examine the energetics and kinetics of the Lys-POPE interaction with a simplified single-bond Arrhenius-Bell model [34], as a first order approximation of the overall lipid-protein binding. In this model, the dissociation is governed by the height of the free-energy barrier in the absence of force, 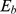, and by the distance between the equilibrium and the transition states, 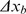. Application of a constant force, 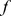, tilts the energy landscape and lowers the barrier, which accelerates the dissociation process. In the presence of a pulling force, the rate of dissociation is given by 
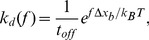
(1)where 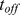 is the spontaneous dissociation time
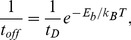
(2)which can be further related to 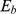 and the diffusive relaxation time (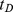, the inverse attempt rate of escape).

**Figure 4:**
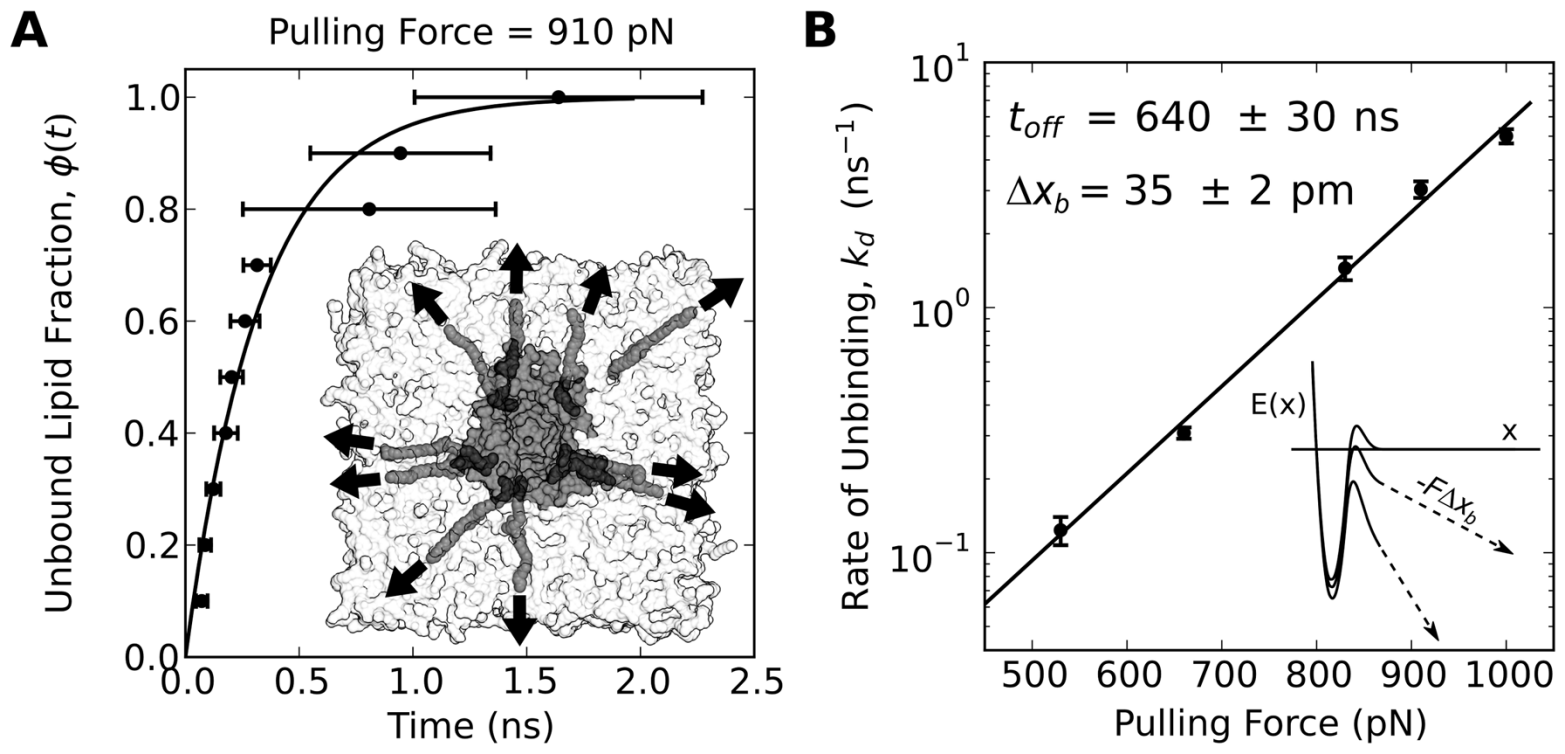
Estimating the strength of lipid binding. (A) Fraction of unbound lipids as a function of time, 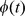, obtained by applying a radial pulling force (910 pN) on the lipid tails while restraining the protein atoms (to prevent the protein from unfolding, see Methods). Data are fitted to the function 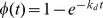 to obtain the rate of unbinding for each pulling force, 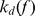. (B) Rates of unbinding are fitted to Eq. 1 to obtain the spontaneous dissociation time (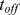) and the distance between the equilibrium and the transition states (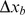).

We characterize the Lys-POPE dissociation of the tightly-bound lipids through non-equilibrium steered MD simulations (see Methods), which may not model any specific biological process but provide kinetic information about bond dissociation. We conduct a series of pulling simulations on the equilibrated membrane-protein system. A radial force is applied simultaneously on the “free” tail of every tightly-bound lipid (10 total), while restraining the protein atoms with harmonic potentials in order to prevent unfolding of the protein due to the large pulling. At a given force, the lipid unbinding events are recorded to obtain the fraction of unbound lipids (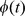) versus time as shown in Fig. 4A for a pulling force of 910 pN. Unbinding of the tightly-bound lipids was determined by the rapid jump in the lipid-protein distance, which was measured for multiple replicate simulations (see Methods for details). Because the likelihood of overcoming the energy barrier in the presence of a pulling force decreases exponentially with the magnitude of the force, we apply very large forces (500–1000 pN) to observe many unbinding events during a reasonable simulation time (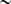
10 ns).

As the lipid headgroups dissociate from the protein lysine residues due to the pulling force, the fraction of unbound lipids, 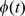, increases over time until they have all dissociated from the protein. Since at high force detached lipids rapidly drift away from the protein, we neglect the rate of association, and therefore 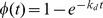. Fitting the unbound lipid fraction to this equation yields a 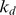 for each pulling force as shown in Fig. 4B. The dependence of 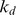 on 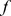 is in good agreement with the Arrhenius-Bell model, and fitting the data in Fig. 4B to Eq. 1 results in 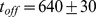 ns and 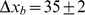 pm. Within the Arrhenius-Bell model, 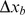 is interpreted as the distance from the equilibrium state of the bond in the absence of force to the location of the activation energy barrier. Simulation and experimentally obtained values of 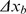 for the dissociation of hydrogen bonds during the unfolding of proteins or peptides can range from 2 to 175 pm [35]–[37], suggesting that there is significant variability in the dissociation stretch. In the case of the Lys-POPE interactions, the hydrogen bond lengths observed in the equilibrium simulation form a narrow distribution as shown in the probability density of P-N distances (Fig. S3), with a full-width at half-maximum value of only 26 pm. Consistently with the predicted Arrhenius-Bell dissociation distance, stable bonds tightly distribute around their median and rarely visit configurations with a 35 pm stretch (Fig. S3). Estimating the attempt rate of escape in the 1–20 ps range [38], the energy barrier (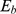) is in the range 10–13 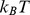.

The obtained spontaneous dissociation time of 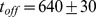 ns is in good agreement with the Lys-POPE kinetics shown in Fig. S1, as we observe many binding and unbinding events during the 500 ns simulation. However, this 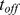 should be interpreted as a lower bound of the spontaneous dissociation time of the whole lipid: even if a Lys-POPE hydrogen-bonding interaction transiently dissociates, because of the confinement of the lipid tail inside the protein cavities, the lipid will remain for a longer time in close proximity of the protein binding site and will likely rebind. In fact, we observe several occurrences of Lys-POPE rebinding in tightly-bound lipids (Fig. S1), which illustrates how the collective molecular features of this lipid-protein interface produce an interaction of remarkable resilience.

### Accelerating the Opening of MscL

To accelerate the opening time in MscL, atomistic MD studies have resorted to unrealistically large membrane tensions [15], [39], to directly applied forces to specific atoms in the protein chosen ad-hoc, [40] or to radial biasing potentials [39]. Following our observation of tightly-bound lipids to the Lys residues on the cytoplasmic side of MscL, we adopt an accelerated simulation protocol to scrutinize gating in atomistic MD, which focuses on these lipid-protein interactions and applies forces directly to the lipids instead. This approach is advantageous because the external forces are not acting directly on the protein atoms, which minimizes the bias of choosing how forces interact with the protein. Furthermore, our results above suggest that these lipids mediate in the force transmission at the lipid-protein interface, acting as firm handles used by the membrane to pull on MscL. Also, by changing the direction of the force, one can explore a space of mechanical stimuli analogous to those caused by hydrophobic mismatch or lateral tension. As discussed in the first section, our traction results suggest a connection between hydrophobic mismatch and forces perpendicular to the membrane (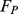, pointing towards the bilayer midplane in the case of positive hydrophobic mismatch), as well as between membrane tension and radially-acting lateral forces (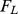).

**Figure 5:**
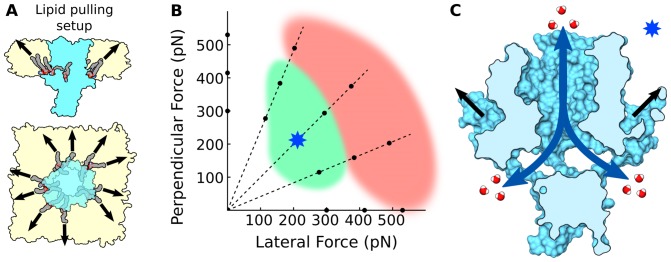
Opening MscL by pulling on tightly-bound lipids. We apply balanced radial pulling forces of different magnitudes and polar angles on the unbound tails of all ten tightly-bound lipids simultaneously as illustrated in (A). In these simulations there are no forces or restraints acting directly on the protein. The diagram in (B) shows regions in the lateral/normal force space for which the channel pore continuously opens under symmetric pulling (all lipids bound, green area), and where the pore opens after one or more lipids unbind and the pulling becomes asymmetric (pink area). Lower forces can readily open the pore, without subunit dissociation, when the lateral and vertical components are close in magnitude. Large forces can result in open conformations with large deformations and partial unfolding/dissociation of the protein subunits in most directions tested. (C) Vertical cross section of a surface representation of the MscL channel opened by a pulling force of 300 pN at an angle of 45°. The C-terminal bundle in this open structure remains associated in good agreement with recent experimental observations [41], [42].

We conduct a series of steered simulations on the equilibrated system, were protein-bound lipids are pulled away from the protein by radially symmetric (in the plane of the membrane) forces at a given polar angle (with respect to the direction perpendicular to the membrane plane) as shown in Fig. 5A (see also Methods). Note that these simulations differ from those used in the previous section, as there are no position restraints nor any other external forces applied directly to the protein. Three force magnitudes are considered (300, 415, and 530 pN) and five polar angles (0, 22.5, 45, 67.5 and 90°), with 0° corresponding to a purely lateral force and 90° corresponding to a purely perpendicular force. The diagram in Fig. 5B delimits the regions in the force plane where 1) the MscL pore gently opens and remains open under symmetric pulling (area shaded in green), and 2) where the pore only opens after one or more lipids dissociate from the protein, the pulling becomes asymmetric, and the protein is severely distorted (area shaded in pink). This diagram was obtained by characterizing the pore diameter, passage of water molecules, lipid unbinding, and subunit dissociation of each of the 15 simulations (see Fig. S4). For the lower pulling forces, the channel selectively opens under certain combinations of lateral and perpendicular forces. Application of lateral and perpendicular forces of equal magnitude, analogous to positive hydrophobic mismatch accompanied by lateral tension, produces the most effective and gentle way to open the MscL pore (when a force of 415 pN at an angle of 45° was applied, the pore began to open in less than 3 ns). Fig. 5C shows a vertical cross section of a surface representation of the channel opened by applying a force of 300 pN to the tightly-bound lipids at an angle of 45°. This structure is characterized by a large central pore in the trans-membrane (TM) region of the protein, which continues onto five openings formed by the stretched loop that links the TM helices with the C-terminal bundle; this helical bundle, often ignored in other computational studies, remains associated in the open structure. Interestingly, recent experimental results have shown that the helices in this bundle remain in very close proximity when MscL is opened by tension [41], and molecular sieve experiments have demonstrated that decreasing the length of the loop that links the TM helices with the C-terminal bundle reduces the conductance and pore size of the protein [42]. Application of large forces (pink region in Fig. 5B) also results in open configurations of the channel, yet these structures typically show partially unfolded/dissociated subunits both in the TM region as well as in the C-terminal bundle (Fig. S5A). While we have only conducted one simulation at each pulling condition, the compiled data shown in Fig. S4 clearly indicates that forces which are purely lateral (Fig. S5B) or purely perpendicular (Fig. S5C) are significantly less effective at opening the channel. We did not succeed in opening MscL at lower forces during a reasonable simulation time (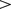
200 ns) due to the exponential dependence of the opening kinetics. However, we expect that the selectivity of gating to force direction will also be present at physiological forces. Thus, MscL appears to be sensitive to the orientation of the forces applied on the tightly-bound lipids, and particularly responsive to mechanical stimuli analogous to combining lateral tension and positive hydrophobic mismatch.

## Discussion

We have presented a simulation approach that combines molecular characterization with a continuum mechanics analysis, which provides insight into how global physical quantities such as membrane tension affect the local behavior within the membrane-protein system. We believe that our results provide a tangible connection between specific molecular features and the transduction of mechanical stimuli. The traction results in the first section identified regions of the protein that are affected by membrane tension, and further provides a conceptual connection, through the separation into normal and tangential components (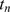 and 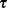), to understand how hydrophobic mismatch and tension change the forces on the surface of the protein. The resilient association of the tightly-bound lipids on the cytoplasmic side of the protein suggests that this lipid-protein complex may play an important role in the force transduction between the bulk membrane and the protein. The N-terminal S1 domain is a dominant player in this association through a specific lipid binding motif. If, according to our hypothesis, this domain is involved in transducing tension to the protein, then we expect that the absence of this motif or amino acid modifications within it will change the channel's sensitivity to membrane tension. Experiments showing that mutations and deletions in the S1 domain can result in MscL channels with decreased or no activity [12], [13], provide strong support for this hypothesis. A more recent study aimed at characterizing the effect of individual cysteine substitutions in the S1 domain of *Escherichia coli* MscL [11] presents observations with opposite behavior (K5C and R8C mutants have increased membrane sensitivity), although the cysteine mutations can produce many unwanted side-effects that could have affected the behavior of the membrane-protein system.

### Possible New Experiments and Revisiting Existing Ones

To further ascertain the role of the amphipathic S1 domain and the nearby lysines in the force transduction of MscL, we suggest some possible experiments. Individual and multiple replacement of the positively charged lysines (K3, K6, K99, and K100) with small neutral amino acids such as alanine may result in considerably weaker anchoring of MscL to the lipid bilayer and therefore increase the membrane tension gating threshold. A similar MscL phenotype may be observed by disrupting the amphipathic nature of the S1 helix, either by making it completely hydrophobic or hydrophilic. Furthermore, direct attachment of lipids to the S1 domain (e.g., through single cysteine mutations in combination with thiolated lipid headgroups) may result in MscL channels with increased sensitivity and a lower membrane tension gating threshold. Such protein modifications may also be studied with simulations, and in particular with the traction analysis we have presented.

Insertion of the lipid tails into the cavities formed by the protein may be modulated by mutation of hydrophobic residues such as Phe in the cytoplasmic side of MscL with either smaller or bulkier amino acids. Changes in lipid affinity to the protein due to mutations may be characterized with fluorescence quenching assays [32]. We expect that variations in the lipid tail affinity will affect the membrane tension gating threshold. This tail affinity may contribute to the observed increase in the tension needed to gate the channel with longer lipid chain-lengths, as our results and experiments [32] suggest a lower affinity for lipids with chains longer than 16 carbons.

The presence of increased lipid interactions on the cytoplasmic side of MscL imposes an asymmetry in the gating process that may have important consequences. The directionality of MscL with respect to the membrane has not been conclusively addressed by in-vitro experimental studies, and there is conflicting evidence regarding the sidedness of MscL in reconstituted liposomes [43], [44]. Directionality of MscL in the membrane is likely to play an important role in the effects that lysophospholipids (LPs) have on the gating process. LPs have been shown to reduce the tension needed for gating MscL, and at high concentrations can actuate the channel in the absence of tension [7], [8]. Yet, this effect is only observed when the LPs are applied to one side of the membrane [8]. Protein directionality with respect to the asymmetric application of LPs has been suggested by experimental evidence on mammalian MS proteins, including the K2P1 channel, showing that the effect of LPs on gating depends on whether they are incorporated on the cytosolic or the periplasmic side of the membrane [45], [46]. Patch-clamp experiments of the effects of lysolipids or other amphipaths may need to be revisited while carefully monitoring the orientation of MscL in the membrane in order to test the effect of this sidedness. The effects of lysolipids should also be studied in combination with the S1 domain mutations suggested above, as this may help understand the origin of the assymetry.

### A Simple yet Robust Membrane Anchoring Motif

**Figure 6:**
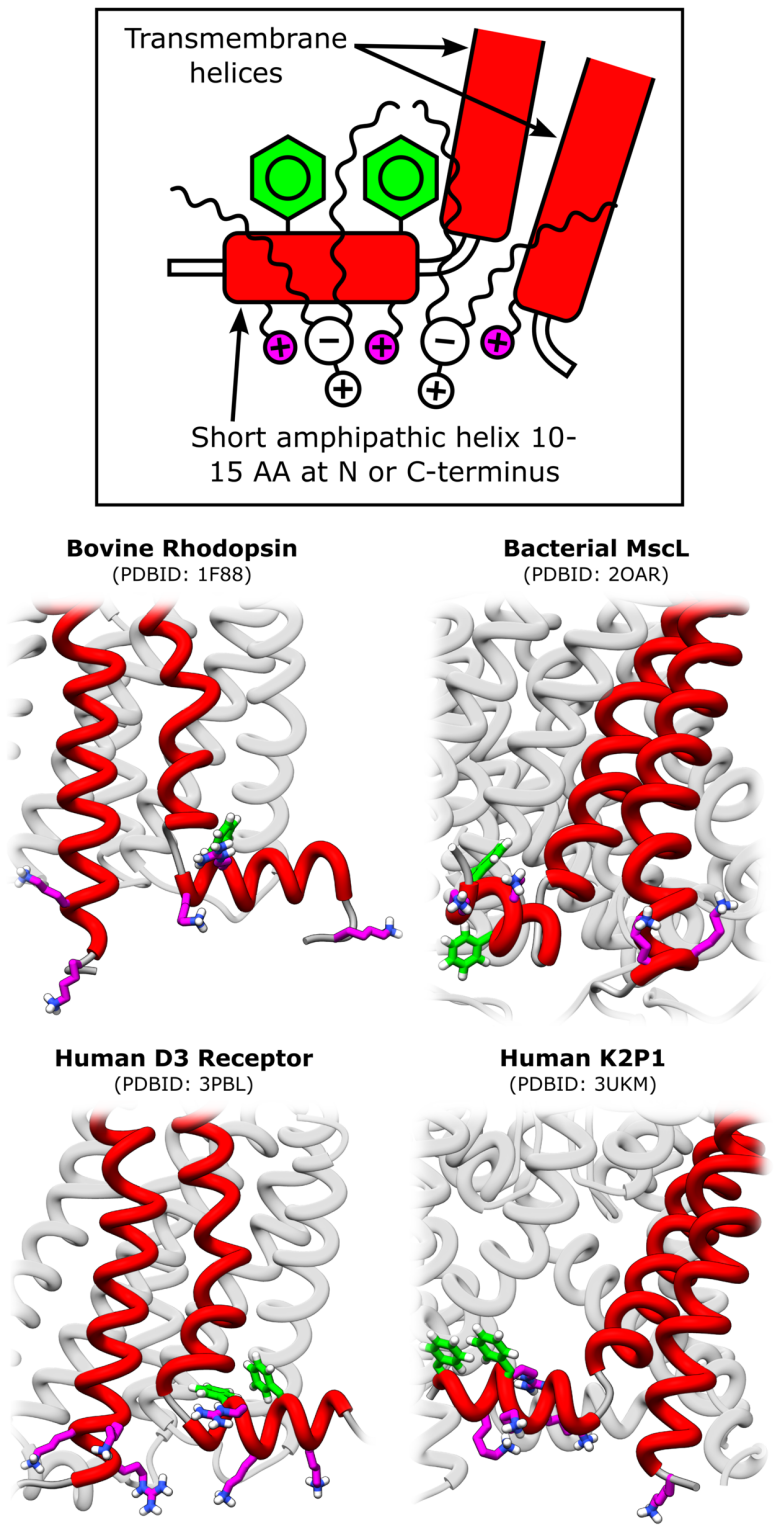
A common motif for phospholipid binding. Examination of the structure of integral membrane proteins including MscL, the human mechanosensitive K2P1 channel TWIK-1 [47], the photo-sensitive G protein-coupled receptor bovine rhodopsin [48], and the human dopamine D3 G protein-coupled receptor [50]. These proteins show the occurrence of a common structural motif, where phospholipids may bind and anchor the protein to the bilayer. The lipid binding motif is composed of a short amphipathic helix (10–15 amino acids) at either the N or C-terminus, which is followed by a sharp bend and a trans-membrane helix. The short amphipathic helix typically contains one or more closely spaced Phe residues on the hydrophobic face, and multiple Lys and sometimes Arg (positively charged) residues on the hydrophilic side.

The observation that the structural motif in the S1 domain appears to be highly conserved in MscL between species [11] may suggest that this motif plays an important and common role in the interaction with the membrane. Indeed, the recently determined structure of the human MS channel K2P1 (TWIK-1) shows an amphipathic helical domain at the C-terminus of remarkable structural similarity to the S1 domain in MscL, and which is thought to affect gating of the channel [47]. We have further identified (non-exhaustively) several other integral membrane proteins that belong to the G protein-coupled receptor (GPCR) family, including bovine rhodopsin [48], [49], the human dopamine D3 receptor [50], and the human serotonin receptor 5-HT1B [51], which contain a common structural motif with two key features: 1) a short amphipathic helix of 10–15 amino acids at the N or C terminus, which is followed by a sharp bend and a TM helix, and 2) a cluster of positively charged residues (Fig. 6). The amphipathic helix contains one or more closely spaced Phe (a bulky, hydrophobic residue) on the hydrophobic face, and multiple Lys and sometimes Arg on the hydrophilic side as well as in nearby trans-membrane helices. The positively charged amino acids provide an interaction site for the electro-negative oxygens in the lipid headgroup, and the hydrophobic residues further increase lipid-protein association by trapping the lipid tail. The structure of some of these proteins, highlighting the structural motif, is shown in Fig. 6.

This lipid-protein association motif provides a simple, yet robust interaction that can strongly anchor a protein to the lipid bilayer. In the case of MscL, our results strongly suggest that this motif is a fundamental component of the force-transduction between the membrane and the protein, and it may play a similar role in other MS channels. Strong anchoring to the membrane would also assist in the tilting of the first TM helix during gating and stabilizing the open channel structure as has been previously proposed [11]. While some of the proteins that we have identified are not directly categorized as mechanosensitive, strong anchoring to the membrane would likely confer some sensitivity to the mechanical state of the membrane. An example of such effect is rhodopsin, which is highly sensitive to membrane curvature and other mechanical properties [49], [52].

While throughout the paper we have emphasized the current limitations of atomistic MD to study channel gating, the protocol we have employed to actuate MscL, applying forces to the tightly-bound lipids, 1) enables us to accelerate the channel opening in a gentle manner, and 2) provides a framework to understand how the mechanical state of the membrane may influence the protein. A force state on the protein-bound lipids, given by a lateral and perpendicular force as shown in Fig. 5, can be interpreted as a proxy for a stress state imposed by mechanical stimuli such as tension, lipid composition, or hydrophobic mismatch. Specific regions in the stress space more easily excite opening, while other regions find a large resistance, which may be interpreted as a selection of gating stress states dictated by the protein free energy landscape, and transmitted by the protein-bound lipids. This interpretation provides a possible connection between gating models relying uniquely on either the bilayer deformation free energy or the protein physics. Although we have placed a high emphasis on the lipids bound to the S1 domain, guided by our continuum analysis of the stress state in the system and by the identification of tightly-bound lipids, we expect that the interactions on the periplasmic side of the protein will also play an important role in the mechanical transduction of forces. The traction data (Fig. 2) showing increased forces on the periplasmic rim of the protein, supports the idea that this region may also be involved in the transduction of forces and tilting of the TM helices as suggested by recent experimental observations [14], [53].

In summary, our results suggest further experimental and theoretical studies on MscL and other membrane proteins, closely examining the directionality with regards to membrane asymmetry, the prevalence of lipid binding for different bilayer compositions or lipid headgroups, and further investigating the role of specific sites at the bilayer-protein interface in the transduction of forces.

## Methods

### System Setup and Equilibration

All simulations were conducted with the GROMACS 4.5.5 simulation package [54] at the Barcelona Supercomputing Center. The membrane-protein system was simulated using a combination of the Gromos 43A1-S3 force field (FF) [55] for the lipids and the more recent Gromos 54A7 FF [56] for the protein. A classical leap-frog integrator was used with a time step of 2 fs. Lennard-Jones interactions where calculated using a twin-range cut-off scheme of 1.0/1.6 nm, with interactions within 1.0 nm updated at every time step and interactions between 1.0 and 1.6 nm only updated every 5 time steps. The particle-mesh Ewald (PME) method was used for computing electrostatic interactions beyond a real-space cut-off of 1.0 nm with a Fourier grid spacing of 0.15 nm. A Nose-Hoover thermostat was used to maintain a constant temperature of 37°C, and a Parrinello-Rahman barostat was used to semi-isotropically (pressures in the membrane plane are coupled) maintain the pressure at 1 atm. A membrane patch composed of 512 POPE (1-palmitoyl-2-oleoyl-sn-glycero-3-phosphoethanolamine) lipids (16 by 16 lipids in each monolayer) was first equilibrated for 400 ns before embedding the protein. The starting MscL structure from *Mycobacterium tuberculosis* (PDBID 2OAR [5]) was first processed to add missing hydrogens followed by energy minimization. This structure was then aligned with the membrane and embedded into the patch by using the *g_membed* procedure [57], which resulted in the deletion of 32 lipids (16 from each leaflet) to accommodate the protein. Estimation of the cross sectional area of MscL using the CHARMM-GUI web service [58], [59] indicates that the area of the protein in both leaflets is in close value, with posible variations in the order of one lipid molecule depending on slight changes in orientation. The large membrane size of our system (480 lipids after embedding, compared to 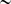
300 used in previous MscL simulation studies [15], [17], [39], [40]) is expected to accommodate any variations in the cross-sectional area of the protein during simulation. The embedded protein system was hydrated with 40,000 water molecules to minimize the interaction of the protein with itself through the periodic boundary. After energy minimization and a short pre-equilibration period of 100 ps with constrained (force constant of 1000 kJ/mol·nm^2^) protein positions and constant volume, the hydrated system was simulated for 500 ns at a constant pressure (1 atm) and temperature (37°C). The equilibration of the system as a whole is relatively slow, taking more than 350 ns for the protein backbone RMSD and the system area to stop drifting. In contrast, the equilibration time scale for an initially folded aqueous protein of a similar size can be in the order of tens of nanoseconds and a fluid membrane patch can reach a steady state in 50–100 ns.

### Local Stress and Traction

After the 500 ns equilibration period, two systems were further simulated for local stress calculations. The first system, in a tensionless state, was a simple extension of 100 ns with the same simulation parameters. For the second system, in a tensioned state, the global pressure in the membrane plane was set to −20 bar and all other parameters remained the same. The area of this system reached a steady-state after 40 ns (reaching an area expansion of 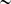
6%) and was continued for a subsequent 100 ns of data collection. Positions and velocities for both systems were collected every 5 ps.

The local stress tensor (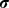) was calculated from the pair-wise particle forces using the central force decomposition methodology [25], [26]. In this method, the simulation volume is divided into uniform blocks and the local stress tensor is calculated for each block from the kinetic and potential contributions 
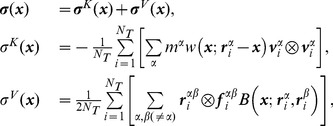
(3)where 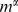, 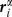, 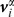 and 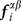 are the masses, positions, velocities, and pairwise forces at time-step 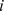, and 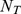 is the total number of time-steps. The symbol 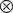 denotes the dyadic product, e.g., 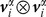 is a second-order tensor. The functions 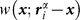 and 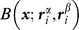 are weighting functions that spatially average the point-wise contributions onto the discretized volume (see Vanegas et al. [25] for a more detailed discussion). While long-range electrostatic interactions were computed using the PME method during the simulation, electrostatic contributions to the local stress were calculated using a plain cut-off with a radius of 2.2 nm due to limitations in the method [25]. The local stress field was calculated using a grid spacing of 0.1 nm. This field was then processed by a gaussian filter with a width of 0.8 nm to remove high frequency fluctuations. The surface used to calculate the traction was computed from the time-averaged mass density of the protein in 3D using a custom GROMACS utility. As with the stress, the density field was processed by a gaussian filter with a width of 0.2 nm. The traction vector (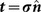) and its components were computed and visualized on an isocontour surface of this filtered density with the program ParaView. Custom programs and scripts for data processing are available to download from http://www.lacan.upc.edu/LocalStressFromMD.

### Lipid Pulling Experiments

In all steered simulations a force of constant magnitude was applied on the center of mass of the lipid tail not bound to the protein. In the simulations used to estimate the strength of the lipid-protein interaction, the pulling direction was parallel to the membrane plane in the radial direction along the vector connecting the center of mass of the protein and the center of mass of the lipid tail. Pulling forces were applied to all ten of the tightly-bound lipids simultaneously. The pulling force, always pulling the lipid away from the protein, continuously changed direction with the system fluctuations. Upon dissociation from the protein, the lipids would become trapped at the simulation boundaries. Given the large pulling forces applied, lipids move away from the protein very rapidly upon dissociation and each unbinding event can be easily determined by a sudden jump in distance between the center of mass of the protein and the center of mass of the lipid. During these simulations, the positions of the protein atoms were restrained using a harmonic potential with force constant of 1000 kJ/mol·nm^2^, which prevented the protein from unfolding or being dragged in the simulation box. At each of the pulling forces (530, 660, 830, 910, and 1000 pN), three to five simulations were conducted and the time for each lipid unbinding event recorded. These times were used to determine the average time required to observe a given fraction of unbound lipids at each force.

In the simulations used to actuate the channel, all ten tighly-bound lipids are also pulled simultaneously and the force on each lipid was defined along a specific vector. These vectors were chosen so that the pulling was uniform and the net force in the membrane plane was balanced. Three pulling forces were used (300, 415, and 530 pN) and five polar angles (0, 22.5, 45, 67.5 and 90°) with respect to the vector perpendicular to the membrane plane. In simulations where the pulling force had a vertical component, a counter-balancing force was also applied onto the center of mass of the periplasmic leaflet (opposite to the bound lipids) to prevent the membrane from bending. For example, if each lipid was pulled with a force of 300 pN at an angle of 45 degrees, the counter-balancing force on the periplasmic leaflet was −300 sin(45°) times the number of lipids (10). In all simulations used to actuate the protein, there were no forces directly acting on the protein nor any position restraints.

### Data Analysis and Visualization

Individual lipid volumes were obtained by computing a radical voronoi tesselation, where the radii of the atoms (estimated from the Lennard-Jones parameters) were used to define the tesselation boundaries over all atoms in the system. Volumes for each molecule were then obtained by summing the volumes of the corresponding atoms. Tesselations were performed with a custom GROMACS utility that uses the VORO++ library [60]. All molecular graphics and visualizations were performed with the UCSF Chimera package [61].

## Supporting Information

Figure S1 **Time traces of the distance between the lipid phosphate atom of the ten tightly-bound lipids and the sidechain nitrogen of cytoplasmic lysine residues of MscL (K3, K6, K99, K100).** In each plot, the blue, green, or red lines show the P-N distance for a selected lipid and the two or three closest Lys residues. The average P-N distance is 0.4 nm when a POPE lipid is hydrogen-bonding a Lys of the protein.
(TIF)

Figure S2
**Average individual lipid volume (A) and variance of the lipid distances with respect to the center of mass of the protein (B) during the equilibration period.** Both of these measurements show significantly decreased fluctuations and lateral mobility of tightly-bound lipids. Lipid binding takes place early in the simulation (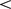
50 ns).(TIF)

Figure S3
**Average distribution of P-N distances for the tightly-bound lipids.** The P-N time traces (Fig. S1) of the closest hydrogen bond (e.g., green line of the first panel in Fig. S1) for each of the tightly-bound lipids were used to create a probability density of observed distances, and then averaged for the 10 lipids. The full-width at half-maximum for the average distribution is 26 pm. The location of the probability density for an extension of 35 pm (corresponding to the fitted value of 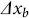) from the median P-N distance is shown for illustration purposes.(TIF)

Figure S4
**Plots, for all tested pulling forces and directions (see subsection 4 of the Results and main Methods), of the average diameter of the pore at the** 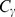 **atom of Val21 (black line), with red triangles indicating instants when a pulled lipid dissociates from the protein, and areas shaded in blue representing the times at which the pore allowed water molecules to pass freely (determined by visual inspection).** Numbers on the left indicate the magnitude of the pulling force on each lipid tail, and the top numbers indicate the polar angle of the pulling force with the respect to the membrane normal (e.g. 0° corresponds to a purely lateral pull).(TIF)

Figure S5 **Cross section of surface representations of MscL opened under different lipid pulling forces and directions (see subsection 4 of the Results and main Methods).** (A) 530 pN at an angle of 22.5°. (B) 415 pN at an angle of 0°. (C) 415 pN at an angle of 90°.
(TIF)
