# Supplementary figures and images for: Force Transduction and Lipid Binding in MscL: A Continuum-Molecular Approach
Source: PLoS One. 2014 Dec 1;9(12):e113947. doi: 10.1371/journal.pone.0113947 (PMC4250078; doi:10.1371/journal.pone.0113947)

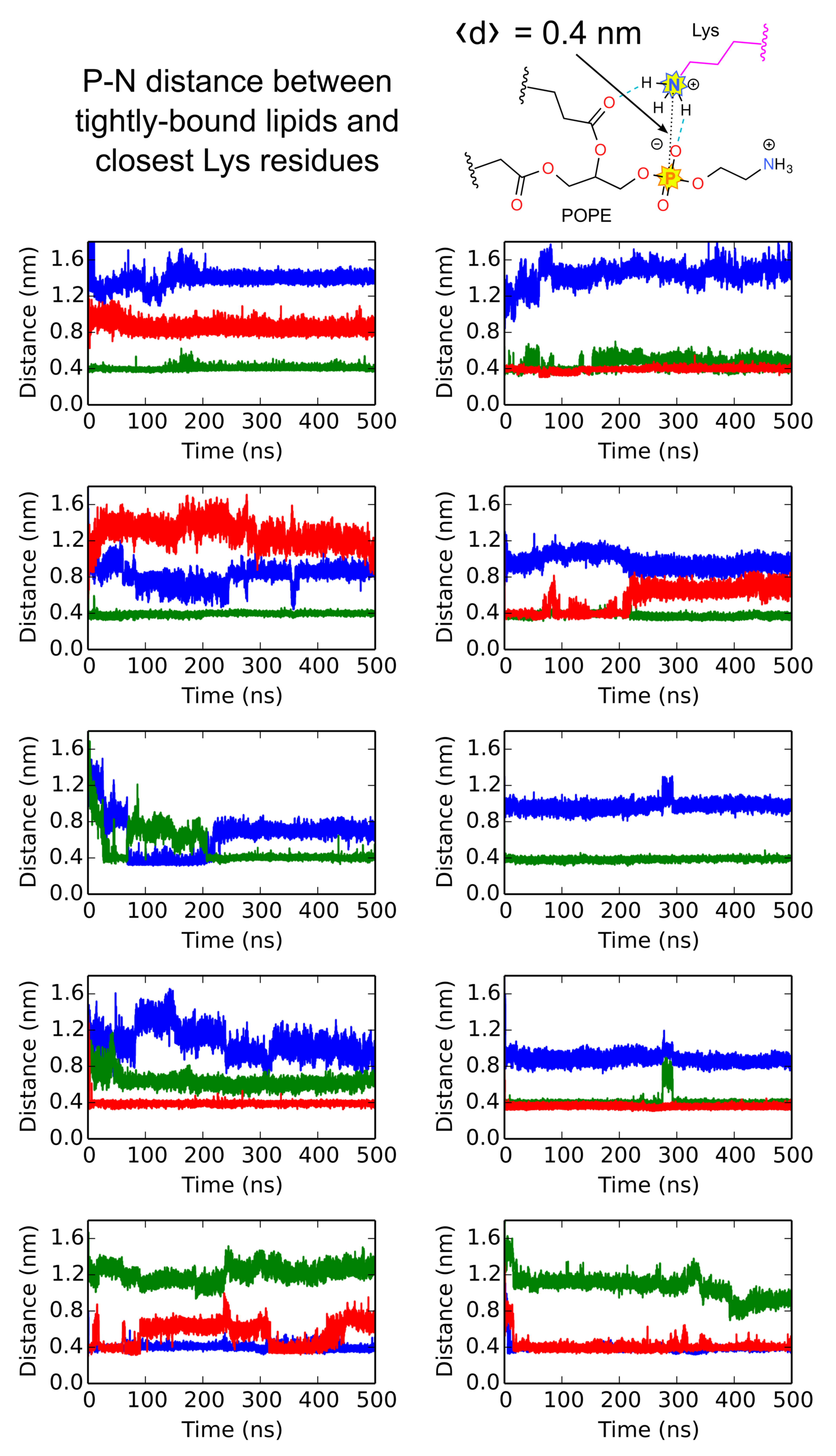

Supplement: Figure S1 — Time traces of the distance between the lipid phosphate atom of the ten tightly-bound lipids and the sidechain nitrogen of cytoplasmic lysine residues of MscL (K3, K6, K99, K100). In each plot, the blue, green, or red lines show the P-N distance for a selected lipid and the two or three closest Lys residues. The average P-N distance is 0.4 nm when a POPE lipid is hydrogen-bonding a Lys of the protein. (TIF) [file pone.0113947.s001.tif]

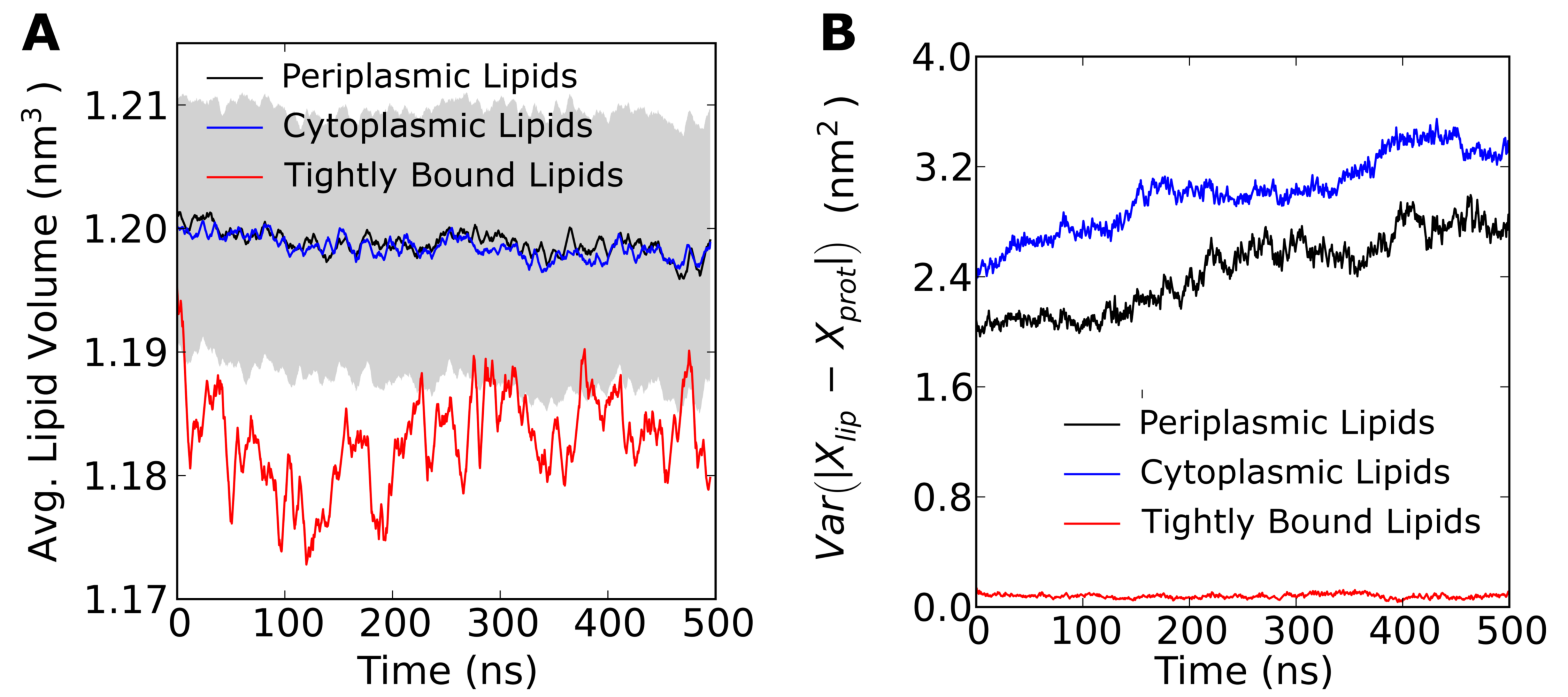

Supplement: Figure S2 — Average individual lipid volume (A) and variance of the lipid distances with respect to the center of mass of the protein (B) during the equilibration period. Both of these measurements show significantly decreased fluctuations and lateral mobility of tightly-bound lipids. Lipid binding takes place early in the simulation (50 ns). (TIF) [file pone.0113947.s002.tif]

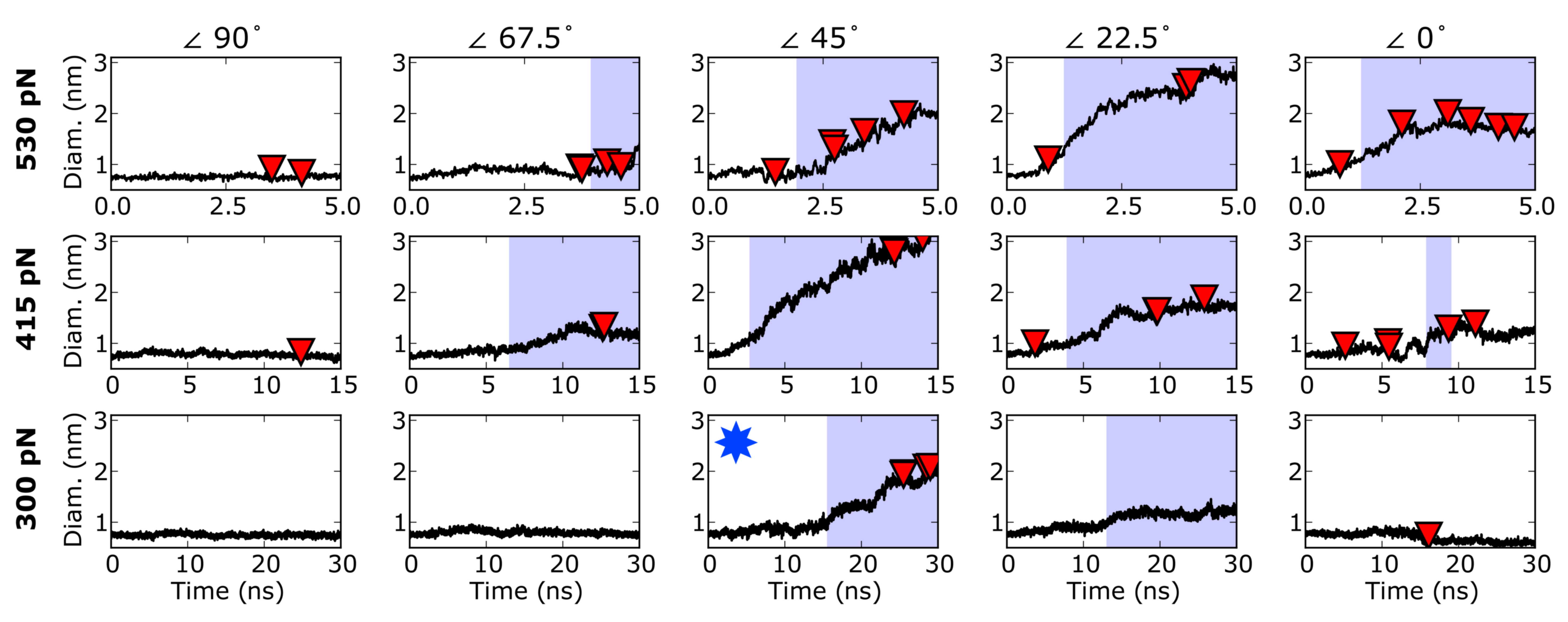

Supplement: Figure S4 — Plots, for all tested pulling forces and directions (see subsection 4 of the Results and main Methods), of the average diameter of the pore at the atom of Val21 (black line), with red triangles indicating instants when a pulled lipid dissociates from the protein, and areas shaded in blue representing the times at which the pore allowed water molecules to pass freely (determined by visual inspection). Numbers on the left indicate the magnitude of the pulling force on each lipid tail, and the top numbers indicate the polar angle of the pulling force with the respect to the membrane normal (e.g. 0° corresponds to a purely lateral pull). (TIF) [file pone.0113947.s004.tif]

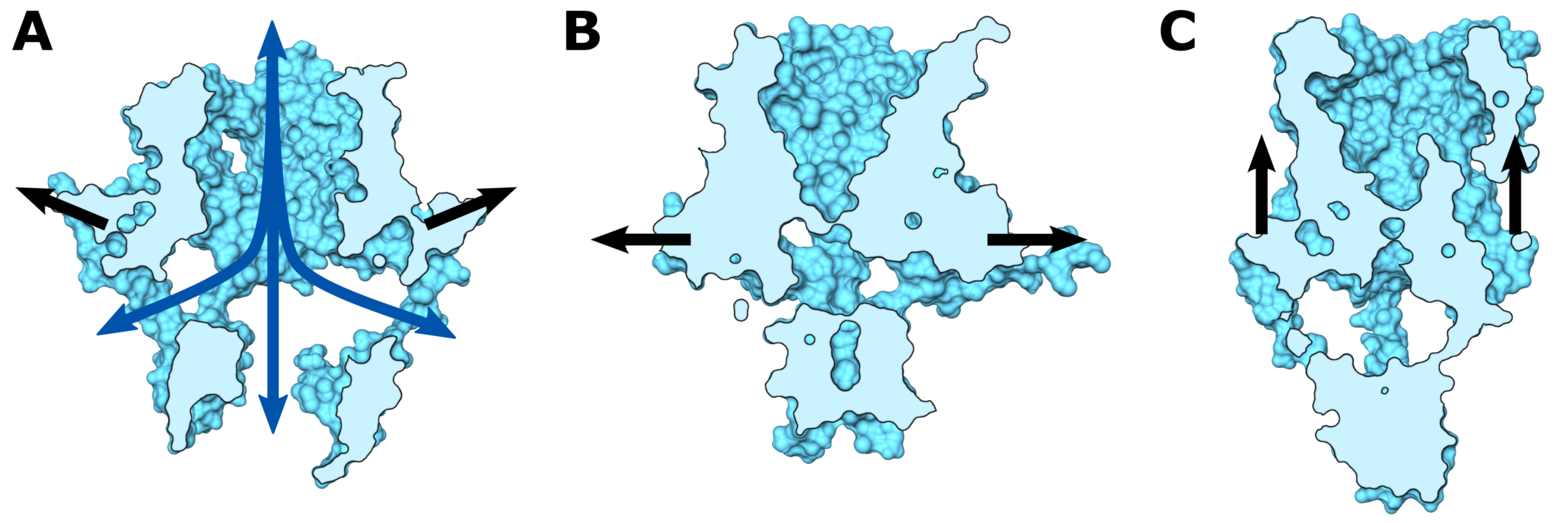

Supplement: Figure S5 — Cross section of surface representations of MscL opened under different lipid pulling forces and directions (see subsection 4 of the Results and main Methods). (A) 530 pN at an angle of 22.5°. (B) 415 pN at an angle of 0°. (C) 415 pN at an angle of 90°. (TIF) [file pone.0113947.s005.tif]
